# Antibiotic prophylaxis in trauma and orthopedic surgery: Current practices across 36 German hospitals and comparison with recently published national guidelines

**DOI:** 10.1007/s00113-025-01659-7

**Published:** 2025-12-05

**Authors:** Susanne Baertl, Siegmund Lang, Leopold Henssler, Lorenz Huber, Markus Rupp, Frank Hanses, Volker Alt

**Affiliations:** 1 Department of Trauma Surgery, University Hospital Regensburg, Regensburg, Germany; 2 Regensburg University Center for Musculoskeletal Infections, University Hospital Regensburg, Regensburg, Germany; 3 Department of Infection Prevention and Infectious Diseases, University Hospital Regensburg, Regensburg, Germany; 4 Department of Trauma, Hand and Reconstructive Surgery, University Hospital Giessen, Giessen, Germany

**Keywords:** Antibiotic prophylaxis, Empirical antibiotic therapy, Local antibiotic therapy, Fracture-related infection, Practice guideline, Antibiotikaprophylaxe, Empirische Antibiotikatherapie, Lokale Antibiotikatherapie, Frakturbedingte Infektion, Praxisleitlinie

## Abstract

**Background:**

Antibiotic prophylaxis is a key component of infection prevention in trauma and orthopedic surgery. Until 2024, no uniform nationwide guidelines existed in Germany regarding the optimal choice, dosage, and duration of perioperative antibiotic use.

**Methods:**

A nationwide survey was conducted among 36 German trauma and orthopedic centers to assess current practices of antibiotic prophylaxis in closed and open fractures, primary arthroplasty, and posterior spinal instrumentation. The questionnaire included the choice and duration of systemic antibiotics, empirical strategies, and the use of local antibiotics such as vancomycin powder. The current practice was then compared to the recently published S3-guideline “Perioperative and periinterventional antibiotic prophylaxis” (AWMF 067-009; https://register.awmf.org/de/leitlinien/detail/067-009) in Germany.

**Results:**

For closed fractures, 94.4% of hospitals used first- or second-generation cephalosporins, with single-shot administration. In Gustilo–Anderson (GA) type I open fractures, cefuroxime and ampicillin/sulbactam were each used by 13 hospitals (36.1%), with 63.9% applying a single-shot regimen. In type III open fractures, piperacillin/tazobactam was most common (33.3%), and 72 h prophylaxis was most frequently reported in both type II and III fractures (38.9%). In distal phalanx fractures, 94.4% of hospitals administered systemic antibiotics despite guideline recommendations to omit prophylaxis when no osteosynthesis is required. In arthroplasty, cefuroxime (50.0%) and cefazolin (41.7%) predominated, with single-shot use in 94.4%. In spine surgery, 38.9% additionally used local vancomycin powder.

**Conclusion:**

While guideline adherence is high in routine indications, significant deviations remain in open fractures and distal phalanx fractures of the fingers. Extended prophylaxis beyond 72 h in GA type III fractures and the frequent use of antibiotics in distal phalanx injuries contrast with current recommendations. Stronger implementation of national standards is essential to reduce overtreatment and support antimicrobial stewardship.

**Graphic abstract:**

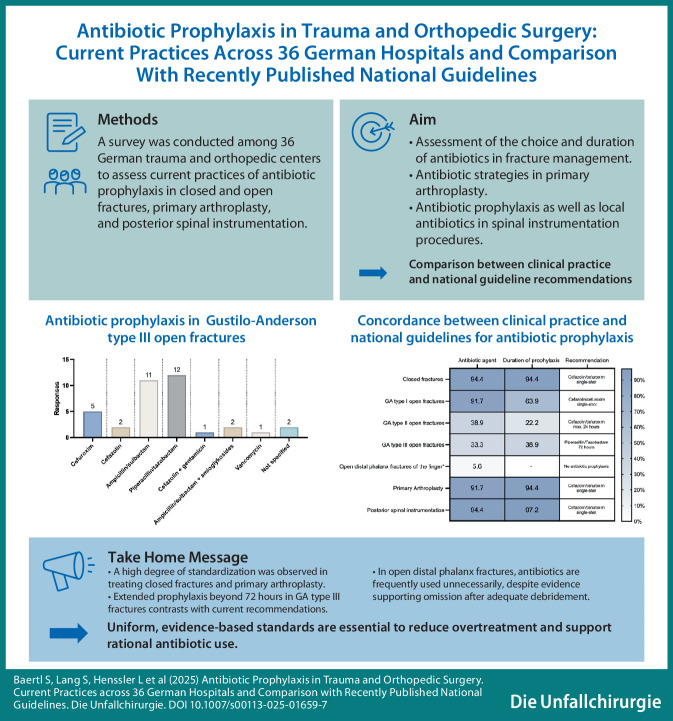

**Supplementary Information:**

The questionnaire used in this study is available in the online version of this article (10.1007/s00113-025-01659-7).

## Abstract (de)

**Hintergrund:**

Die perioperative Antibiotikaprophylaxe ist eine zentrale Maßnahme zur Infektionsvermeidung in der Unfallchirurgie und Orthopädie. Bis 2024 fehlten in Deutschland einheitliche nationale Empfehlungen zur Auswahl, Dosierung und Dauer der Prophylaxe.

**Methode:**

Im Rahmen einer bundesweiten Umfrage wurden 36 unfallchirurgische und orthopädische Kliniken zu ihren aktuellen Strategien der Antibiotikaprophylaxe befragt. Erfasst wurden der Einsatz systemischer Antibiotika, die Dauer der Gabe, empirische Strategien sowie die Anwendung lokaler Antibiotika wie Vancomycin-Pulver. Die erhobene Praxis wurde mit der im Jahr 2024 veröffentlichten S3-Leitlinie „Perioperative und periinterventionelle Antibiotikaprophylaxe“ (AWMF 067-009; https://register.awmf.org/de/leitlinien/detail/067-009) verglichen.

**Ergebnisse:**

Bei geschlossenen Frakturen verwendeten 94,4 % der Kliniken Cephalosporine der ersten oder zweiten Generation, wobei in ebenso vielen Fällen eine Single-Shot-Gabe erfolgte. Bei Gustilo-Anderson(GA)-Typ-I-Frakturen dominierten Cefuroxim und Ampicillin/Sulbactam (je 36,1 %), wobei 63,9 % der Kliniken eine Einmalgabe durchführten. Piperacillin/Tazobactam war bei GA-Typ-III-Frakturen am häufigsten (33,3 %); eine 72-h-Prophylaxe wurde bei GA-Typ II und III jeweils von 38,9 % der Kliniken durchgeführt. Bei offenen Fingerendgliedfrakturen gaben 34 von 36 Kliniken eine systemische Prophylaxe an – trotz Empfehlung zum Verzicht bei adäquatem Débridement und fehlender Osteosynthese. In der Endoprothetik dominierten Cefuroxim (50,0 %) und Cefazolin (41,7 %), mit 94,4 % Einmalgaben. Bei dorsalen Wirbelsäulenstabilisierungen verwendeten 38,9 % der Kliniken Vancomycin-Pulver lokal zusätzlich zur systemischen Gabe.

**Schlussfolgerung:**

Während bei Routineindikationen eine gute Leitlinienkonformität besteht, zeigen sich bei offenen Frakturen und Fingerendgliedfrakturen deutliche Abweichungen. Insbesondere bei GA-Typ-III-Frakturen ist eine verlängerte Prophylaxe über 72 h hinaus trotz fehlender Evidenz weit verbreitet. Auch bei Fingerendgliedfrakturen erfolgt häufig eine nicht empfohlene Antibiotikagabe. Die Ergebnisse unterstreichen die Notwendigkeit einer konsequenten Umsetzung der neuen S3-Leitlinie zur Vermeidung von Übertherapie und zur Förderung einer rationalen Antibiotikaanwendung.

## Practice implications

- In closed fractures and primary arthroplasty, antibiotic prophylaxis is mostly guideline-compliant, using single-shot cephalosporins.
- A high variability in the treatment of open fractures, especially those classified as Gustilo–Anderson type III, is observed with regard to antibiotic choice and duration.
- In open distal phalanx fractures, antibiotics are frequently used unnecessarily, despite evidence supporting omission after adequate debridement.
- Local antibiotics (e.g., vancomycin powder) are frequently applied in spinal surgery, although current guidelines do not recommend routine use.
- Uniform, evidence-based standards are essential to reduce overtreatment and support rational antibiotic use.

## Introduction

Fracture-related infections (FRI) and periprosthetic joint infections (PJI) remain among the most serious complications in orthopedic and trauma surgery. While infection rates in closed fractures range from 1 to 2%, they can reach up to 30% in type III open fractures according to the Gustilo-Anderson (GA) classification [2]. In arthroplasty, the German Arthroplasty Registry (Endoprothesenregister Deutschland; EPRD) continues to report increasing numbers of revision surgeries due to PJIs, despite strict aseptic protocols [17]. Perioperative antibiotic prophylaxis has become a widely accepted standard to prevent such infections. Data from 8447 participants across 23 studies demonstrated a significant reduction in deep and superficial surgical site infections, as well as urinary and respiratory tract infections, following prophylactic antibiotic administration in the surgical management of closed fractures [7]. Despite the clinical importance, national guidelines on antibiotic prophylaxis in trauma and orthopedic surgery in Germany have been limited until recently. A previous survey revealed substantial discrepancies in the choice of antibiotic agents used for prophylaxis in both closed and open fractures [3]. However, no systematic data have been available regarding the duration of antibiotic prophylaxis or clinical practice in spinal instrumentation procedures. In 2024, nationwide guidelines for perioperative and peri-interventional antibiotic prophylaxis with the “S3-Leitlinie ‘Perioperative und periinterventionelle Antibiotikaprophylaxe’” were finally introduced in Germany [1]. This study was conducted just before implementing these national recommendations and aimed to systematically evaluate current clinical practices regarding antibiotic prophylaxis across German orthopedic and trauma centers. Using a structured, nationwide survey, we assessed the selection of systemic and local antibiotics as well as the duration of prophylaxis in different clinical scenarios, including closed and open fractures, primary arthroplasty, and posterior spinal instrumentation. The answers were then compared to the recently published S3 guideline to identify important differences between the current practice and the guidelines.

## Methods

### Questionnaire

A questionnaire (in German) consisting of 15 targeted questions (Suppl. 1) was developed to assess the current practices in antibiotic prophylaxis across German hospitals. The questionnaire addressed the preferred antibiotic agents utilized in various clinical scenarios, including closed and open fractures, primary arthroplasty, and dorsal spinal instrumentation procedures. Open fractures were categorized according to the Gustilo–Anderson (GA) classification into types I–III, allowing precise identification of the antibiotic strategies employed for each severity grade. Antibiotic prophylaxis strategies specifically for open distal phalanx fractures of the fingers were evaluated through a dedicated question.

In addition, the questionnaire also examined the duration of antibiotic prophylaxis or empirical antibiotic treatment separately for each clinical scenario—closed fractures, open fractures (GA types I–III), primary arthroplasty procedures, dorsal spinal stabilization surgeries, and open distal phalanx fractures.

Moreover, the survey assessed the specific use of local antibiotics by including a binary question (yes/no) on the intraoperative application of vancomycin powder in dorsal spinal stabilization surgery. All remaining questions were designed as open-ended, allowing respondents to freely specify their preferred antibiotics and treatment durations. Respondents were asked to indicate the treatment durations in hours since the occurrence of trauma or the time of surgery.

### Survey

The questionnaire was digitalized using Google Forms (Google Docs Editors Suite) to facilitate easy distribution and data collection. It was sent electronically to all German university hospitals and clinics specialized in trauma and orthopedic care. To ensure anonymity and unbiased responses, the questionnaires were completed anonymously, thereby fully blinding investigators to the respondents’ identities and institutions. Overall, 36 clinics participated and fully completed the questionnaire.

### Statistics

The descriptive and statistical data analysis was performed using GraphPad Prism software (version 10.4.2).

## Results

### Antibiotic prophylaxis in fracture management

**Fig. 1:**
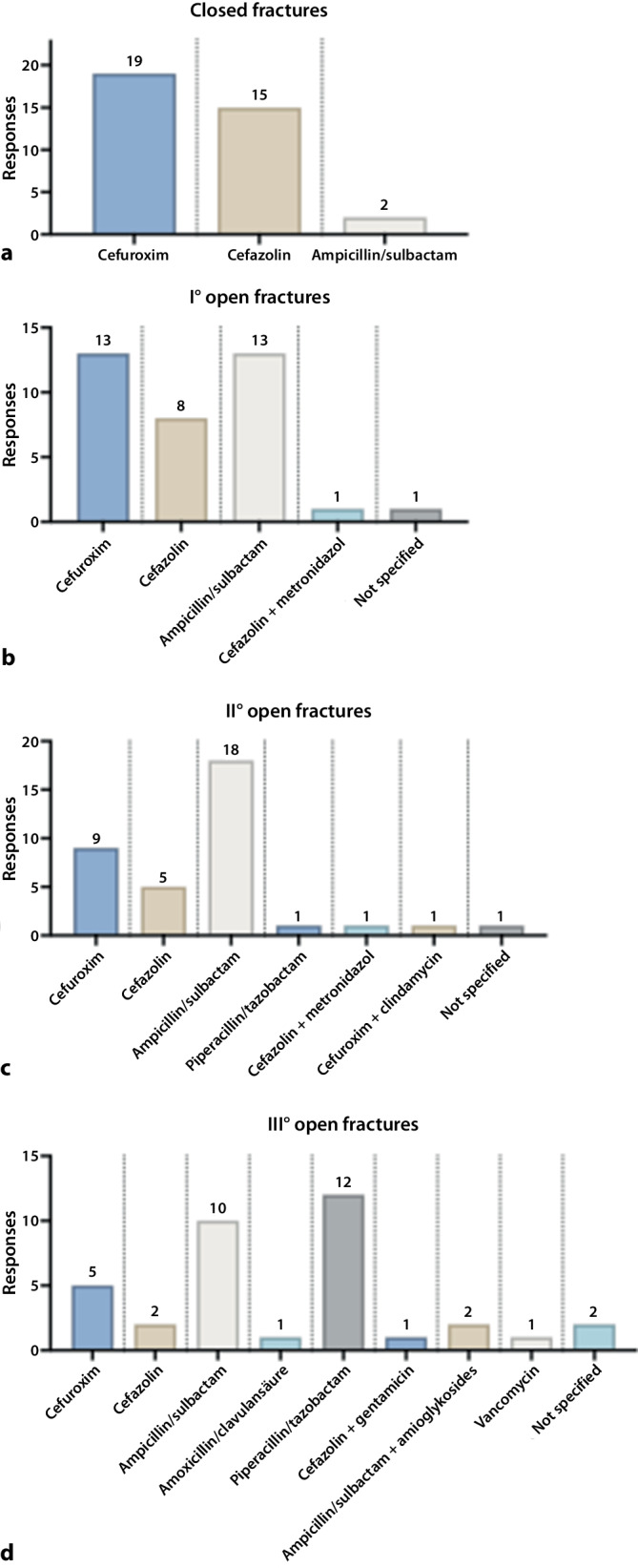
Antibiotic prophylaxis in **a** the surgical management of closed fractures, **b** Gustilo–Anderson (GA) type I open fractures, **c** GA type II open fractures, and **d** GA type III open fractures

In the surgical management of closed fractures, the vast majority of participating hospitals (94.4%) reported using first- or second-generation cephalosporins for antibiotic prophylaxis. Among these, cefazolin was selected by 15 hospitals (41.7%). Cefuroxime represented the standard choice in 19 hospitals (52.8%) (Fig. 1a). Regarding the duration of prophylaxis, a single-shot administration was the predominant practice (94.4%). In contrast, two hospitals indicated a standard duration of prophylaxis extending to 24 h (Table 1).

**Table 1:** Duration of antibiotic prophylaxis in the management of closed and Gustilo–Anderson (GA) type I–III open fractures

	Closed fractures	GA type I open fractures	GA type II open fractures	GA type III open fractures
Single-shot	34 (94.4%)	13 (36.1%)	4 (11.1%)	2 (5.6%)
Two administrations	0	1 (2.8%)	0	0
24 h	2 (5.6%)	10 (27.8%)	8 (22.2%)	1 (2.8%)
48 h	0	2 (5.6%)	2 (5.6%)	1 (2.8%)
72 h	0	4 (11.1%)	14 (38.9%)	14 (38.9%)
120 h	0	3 (8.3%)	2 (5.6%)	7 (19.4%)
144 h	0	0	1 (2.8%)	1 (2.8%)
7 days	0	1 (2.8%)	2 (5.6%)	3 (8.3%)
10 days	0	0	0	1 (2.8%)
14 days	0	0	0	1 (2.8%)
Other*	0	2 (5.6%)	3 (8.3%)	5 (13.9%)

*These cases included prolonged treatment until wound healing was completed or 2 weeks after wound healing was completed, until microbiological results were obtained, or not specified

In the management of open fractures, no clear standard clinical practice could be observed, with a wide variety of antibiotic prophylaxis regimes. In the management of GA type I open fractures (Fig. 1b), nearly all participating hospitals favored the use of first- or second-generation cephalosporins or ampicillin/sulbactam for antibiotic prophylaxis. Cefuroxime or ampicillin/sulbactam were the most frequently chosen antibiotic agents, reported by 13 hospitals each (36.1%), followed closely by cefazolin, selected by 8 hospitals (22.2%). Regarding prophylaxis duration (Table 1), a single-shot administration was again the predominant standard (36.1%), although an extended duration of 24 h was reported by 10 hospitals (27.8%).

In the treatment of GA type II open fractures, hospitals reported using a total of seven different antibiotic agents for prophylaxis (Fig. 1c). Ampicillin/sulbactam was the most frequently chosen antibiotic, selected by 18 hospitals (50.0%). Regarding the duration of prophylaxis (Table 1), the most common practice was a 72 h administration (38.9%). A prophylaxis duration of 24 h was reported by eight hospitals (22.2%).

In the antibiotic prophylaxis of type III open fractures, hospitals reported the use of eight different antibiotic agents (Fig. 1d). Piperacillin/tazobactam was the most frequently chosen antibiotic combination (33.3%, 12 hospitals), followed by ampicillin/sulbactam (30.6%, 11 hospitals). Other antibiotic agents were selected less frequently, including clindamycin, aminoglycosides, carbapenems, fluoroquinolones, and cephalosporins. Regarding prophylaxis duration, an administration of 72 h was predominant (38.9%, 14 hospitals). Other durations, such as 120 h up to 14 days, were reported, highlighting a clear tendency toward extended prophylactic regimens in managing these severe injuries (Table 1).

**Fig. 2:**
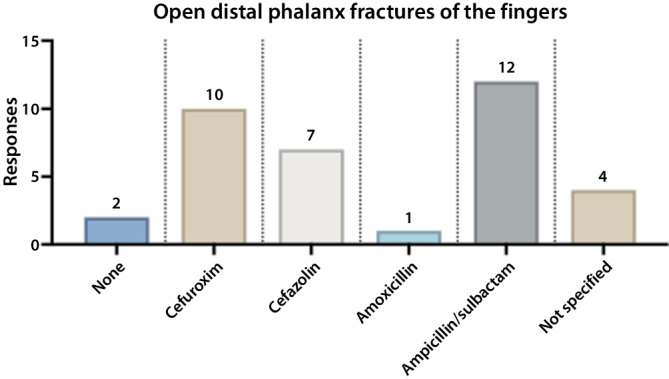
Antibiotic prophylaxis in open distal phalanx fractures of the fingers

We specifically assessed antibiotic prophylaxis practices in the management of open fractures of the distal phalanges of the fingers. Participating hospitals reported the use of six different antibiotic agents (Fig. 2). Aminopenicillins combined with beta-lactamase inhibitors (amoxicillin/clavulanic acid or ampicillin/sulbactam) were the most frequently selected antibiotics (33.3%, 12 hospitals), followed by cefuroxime (27.8%, 10 hospitals) and cefazolin (19.4%, 7 hospitals). Amoxicillin and other agents were chosen less frequently. Regarding the duration of antibiotic prophylaxis for open distal phalanx fractures, single-shot and 24 h regimens were each reported by 7 hospitals (19.4%). A 72 h regimen was the most common, chosen by 9 hospitals (25.0%). A 36 h regimen was used by one hospital (2.8%), a 120 h regimen by 4 hospitals (11.1%), and a 7-day regimen by one hospital (2.8%). In 5.6% of cases (2 hospitals), no antibiotic prophylaxis was administered. In 13.9% of cases (5 hospitals), the duration of prophylaxis was not specified.

### Antibiotic prophylaxis in primary arthroplasty

In primary arthroplasty, a uniform antibiotic regimen was reported across the participating hospitals. Most centers used first- or second-generation cephalosporins for prophylaxis, with cefuroxime being the most frequently selected agent (50.0%, 18 hospitals), followed by cefazolin (41.7%, 15 hospitals). Ampicillin/sulbactam was used less frequently (8.3%, 3 hospitals). Regarding the duration of prophylaxis, a single-shot regimen was reported by most hospitals (94.4%, 34 hospitals), whereas a 24 h regimen was chosen by 5.6% (2 hospitals).

### Antibiotic prophylaxis in posterior spinal instrumentation

In the context of posterior spinal instrumentation, most hospitals reported using first- or second-generation cephalosporins for antibiotic prophylaxis. Cefuroxime was the most frequently selected agent (52.8%, 19 hospitals), followed by cefazolin (41.7%, 15 hospitals). Ampicillin/sulbactam was used less frequently (5.6%, 2 hospitals). Almost all hospitals (97.2%, 35 hospitals) indicated single-shot antibiotic administration, while one hospital preferred antibiotic prophylaxis for 24 h. The use of local antibiotics (vancomycin powder) in addition to systemic prophylaxis was reported by 38.9% of hospitals (14 hospitals), while 61.1% (22 hospitals) did not use prophylactic local antibiotic application.

### Comparison with national guidelines

**Fig. 3:**
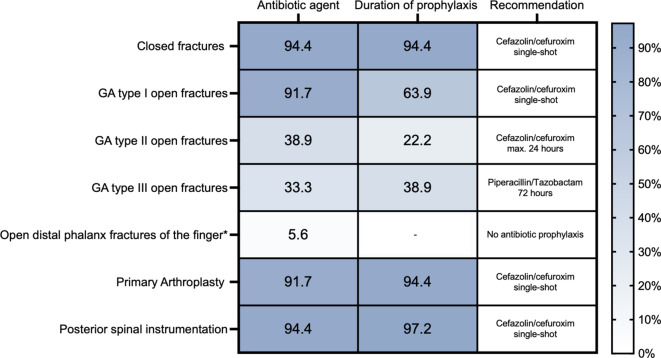
Concordance between clinical practice and national guideline recommendations [1] for antibiotic prophylaxis across common orthopedic and trauma procedures. Percentages indicate hospitals following guideline-concordant agents and duration. GA Gustilo–Anderson. *No antibiotic prophylaxis recommended if no osteosynthesis is required after adequate debridement

Overall, a high level of agreement between current clinical practice and recently published national guidelines [1] was observed in well-established indications such as closed fractures, primary arthroplasty, and posterior spinal instrumentation (Fig. 3). In these areas, more than 90% of hospitals reported using guideline-concordant antibiotic agents, primarily first- or second-generation cephalosporins, and a single-shot regimen. In contrast, significant deviations from current guidelines were observed in the treatment of open fractures and distal phalanx injuries. Particularly, in GA type II open fractures, only 8 of 36 hospitals (22.2%) limited antibiotic prophylaxis to the guideline-recommended duration of 24 h (Fig. 3). Instead, the majority extended the prophylaxis, with 14 hospitals (38.9%) using a 72 h regimen and others reporting even longer durations. In GA type III fractures, the use of piperacillin/tazobactam was reported by 33.3% of hospitals, and 38.9% used a 72 h regimen, while several centers extended the duration up to 120 h or more. The greatest divergence from current recommendations was seen in open distal phalanx fractures of the finger. Despite the S3 guideline explicitly recommending no antibiotic prophylaxis when no osteosynthesis is required and debridement is adequate, 94.4% of hospitals (34 out of 36) reported administering systemic intravenous or oral antibiotics. Only 5.6% (2 hospitals) adhered to the guideline by omitting prophylaxis (Fig. 3). Notably, in 14 hospitals (38,9%), the duration of prophylaxis reached or exceeded 72 h, underlining a clear tendency toward overtreatment in this low-risk indication.

### Discussion

This nationwide survey highlights a remaining variability in antibiotic prophylaxis practices across German orthopedic and trauma centers, particularly in managing open fractures and the local application of vancomycin powder in spinal surgery. It is important to distinguish between peri-interventional prophylaxis (e.g., in closed fractures) and a truly pre-emptive antibiotic approach in contaminated or high-risk situations such as Gustilo–Anderson type II–III open fractures. A high degree of standardization was observed in the peri-interventional prophylaxis of closed fractures and primary arthroplasty. The majority of hospitals adhered to the use of first- or second-generation cephalosporins, with cefuroxime and cefazolin being the predominant agents, and almost all institutions favored single-shot administration. This is consistent with the recently published guideline, which recommend single-shot antibiotic prophylaxis with cefazolin or, alternatively, cefuroxime in primary arthroplasty and the surgical treatment of closed fractures [1]. In contrast, management strategies for open fractures revealed substantial heterogeneity, which was also evident in the nationwide survey conducted in 2022 [3]. Although cephalosporins remained the mainstay for GA type I open fractures, higher-grade open fractures showed an increasing use of broad-spectrum antibiotics, such as aminopenicillins with beta-lactamase inhibitors and piperacillin/tazobactam or even a combination of two antibiotic agents. This seems reasonable as studies revealed an increasing colonization with gram-negative pathogens in the presence of substantial soft tissue damage, especially in GA type III fractures [12, 16]. Therefore, the current guideline recommends piperacillin/tazobactam in those fractures, while cefazolin or cefuroxime remain the first-line antibiotics in GA type I and II fractures [1]. Particularly striking was the variation in treatment duration, ranging from single-shot to prolonged administration of up to 14 days or more. In Gustilo–Anderson type II fractures, only 22.2% of hospitals adhered to the recommended 24 prophylaxis duration, while in GA type III fractures several centers extended the duration up to 120 h or more. This broad range suggests a lack of consensus and highlights the ongoing uncertainty regarding optimal prophylaxis length, especially in complex or contaminated injuries. A systematic review by Chang et al. [5], including four randomized controlled trials with 472 patients, showed a significantly reduced infection rate in open fractures in general. No clear superiority of extended prophylactic regimens (e.g., 3–5 days) over shorter protocols (e.g., 24 h) has been demonstrated for open fractures in general [5]. For GA type III open fractures, however, prolonged antibiotic administration is widely recommended, with the strongest available evidence supporting a 72 h duration [6, 10]. In the present study, more than 50% of the participating hospitals reported prolonged antibiotic prophylaxis exceeding 72 h, likely reflecting attempts to further mitigate infection risk. However, this practice contradicts current evidence, as available trials have shown no clinical benefit and even suggest an increased risk for the selection of multidrug-resistant bacteria in these cases [5, 14].

The greatest divergence from current recommendations was seen in open distal phalanx fractures of the finger, with only 5.6% of hospitals omitting antibiotic prophylaxis. Notably, 25.0% of hospitals reported a prophylaxis duration exceeding 72 h in these low-risk injuries. The current guideline recommends omitting antibiotics in open distal phalangeal fractures that do not require osteosynthesis, provided that adequate surgical debridement is performed [1]. This recommendation is supported by randomized controlled trials showing no increased infection risk in such cases [11]. Especially in times of further increasing antibiotic resistance, a more evidence-based and drug-saving approach would be beneficial [4].

Systemic antibiotic prophylaxis is recommended in dorsal instrumentation of the spine, as this has been shown to decrease the risk for surgical site infections and especially for vertebral osteomyelitis [15]. In the present survey, the vast majority of the hospitals followed the current guideline with single-shot administration of cefazolin or cefuroxime [1]. In spine surgery, the use of local antibiotics, especially vancomycin powder, has become a common practice worldwide. A survey by the AO Spine in the year 2023 [13], as well as our present results, show that over one-third of the hospitals reported the use of local vancomycin powder when performing posterior spinal instrumentation. Although promising results have been reported in the literature regarding reduced SSI rates [9], its use remains controversial and unstandardized in Germany. A meta-analysis by Zale et al. revealed no significant difference in reducing surgical site infections, with 1.45% in the control group compared to 1.26% in the group with local application of vancomycin. Moreover, a shift towards increased rates of gram-negative surgical site infections in patients who received local vancomycin powder during index surgery was reported by Wu et al. [8]. Therefore, the current guidelines refrains from a general recommendation of its use in posterior spinal instrumentation [1].

This study has several limitations. First, the sample primarily consisted of tertiary care hospitals, which may have introduced a selection bias toward more complex cases and a greater tendency for prolonged antibiotic prophylaxis. This focus was intentional, based on the expectation of higher academic engagement and response rates. Second, the study cannot account for specific clinical decisions that may justify deviations from standard protocols. It also remains unclear to what extent the observed heterogeneity reflects patient-related risk factors, trauma mechanisms, or wound contamination, as such parameters were not systematically captured. Third, all data were self-reported and may be subject to reporting bias. No verification of the provided information was conducted. Finally, some open-ended questions, particularly regarding the duration of prophylaxis, may have been interpreted differently by respondents, potentially leading to inconsistency in the responses.

Our study demonstrates high adherence to the national antibiotic prophylaxis guideline in standardized procedures such as closed fractures, arthroplasty, and spinal instrumentation. However, considerable variations remain in the treatment of open fractures and open phalanx injuries. The clearest deviations were observed in Gustilo-Anderson type II and III fractures, where prolonged antibiotic administration beyond 72 h was frequently reported, as well as in open distal phalanx injuries, where prophylaxis was often used despite clear recommendations to omit it. These findings highlight the urgent need to promote uniform implementation of the S3 guideline across all trauma centers to reduce overtreatment, improve patient outcomes, and support antimicrobial stewardship.

## Supplementary Information

The questionnaire used in this study

## Data Availability

The data presented in this manuscript are available from the authors upon reasonable request.

## Declarations

No studies involving human participants or animals were conducted by the authors for this article. For all other cited studies, the respective ethical guidelines as stated in those sources apply.

## References

[1] Abele-Horn M, Novotny A, Bader L et al Perioperative und periinterventionelle antibiotikaprophylaxe (PAP). https://register.awmf.org/de/leitlinien/detail/067-009

[2] Baertl S, Metsemakers W‑J, Morgenstern M et al (2021) Fracture-related infection. Bone Joint Res 10:351–353. 10.1302/2046-3758.106.BJR-2021-0167.R1 34076501 10.1302/2046-3758.106.BJR-2021-0167.R1 PMC8242679

[3] Bärtl S, Walter N, Lang S et al (2022) Antibiotikaeinsatz zu Prophylaxe und empirischer Therapie von frakturassoziierten Infektionen in Deutschland: Eine Umfrage an 44 Kliniken. Unfallchirurgie 10.1007/s00113-022-01200-0 10.1007/s00113-022-01200-0 PMC10450009 35750887

[4] Cassini A, Högberg LD, Plachouras D et al (2019) Attributable deaths and disability-adjusted life-years caused by infections with antibiotic-resistant bacteria in the EU and the European economic area in 2015: a population-level modelling analysis. Lancet Infect Dis 19:56–66. 10.1016/S1473-3099(18)30605-4 30409683 10.1016/S1473-3099(18)30605-4 PMC6300481

[5] Chang Y, Kennedy SA, Bhandari M et al (2015) Effects of antibiotic prophylaxis in patients with open fracture of the extremities: a systematic review of randomized controlled trials. JBVS Rev. 10.2106/JBJS.RVW.N.00088 10.2106/JBJS.RVW.N.00088 27490013

[6] Declercq P, Zalavras C, Nijssen A et al (2021) Impact of duration of perioperative antibiotic prophylaxis on development of fracture-related infection in open fractures. Arch Orthop Trauma Surg 141:235–243. 10.1007/s00402-020-03474-8 32409906 10.1007/s00402-020-03474-8

[7] Gillespie WJ, Walenkamp GH (2010) Antibiotic prophylaxis for surgery for proximal femoral and other closed long bone fractures. Cochrane Database Syst Rev. 10.1002/14651858.CD000244.pub2 11279687 10.1002/14651858.CD000244

[8] Hu W, Wang H, Wu X et al (2023) Does the Microflora of surgery site infection change after prophylactic use of Vancomycin powder in the spine surgery. IDR 16:105–113. 10.2147/IDR.S390837 10.2147/IDR.S390837 PMC9831077 36636373

[9] Luo H, Ren Y, Su Y et al (2022) Intraoperative vancomycin powder to reduce surgical site infections after posterior spine surgery: a systematic review and meta-analysis. EFORT Open Rev 7:109–121. 10.1530/EOR-21-0077 35192507 10.1530/EOR-21-0077 PMC8897567

[10] Messner J, Papakostidis C, Giannoudis PV, Kanakaris NK (2017) Duration of administration of antibiotic agents for open fractures: meta-analysis of the existing evidence. Surg Infect 18:854–867. 10.1089/sur.2017.108 10.1089/sur.2017.108 28956724

[11] Metcalfe D, Aquilina AL, Hedley HM (2016) Prophylactic antibiotics in open distal phalanx fractures: systematic review and meta-analysis. J Hand Surg Eur Vol 41:423–430. 10.1177/1753193415601055 26329883 10.1177/1753193415601055

[12] Otchwemah R, Grams V, Tjardes T et al (2015) Bacterial contamination of open fractures—pathogens, antibiotic resistances and therapeutic regimes in four hospitals of the trauma network cologne, Germany. Injury 46:S104–S108. 10.1016/S0020-1383(15)30027-9 26542854 10.1016/S0020-1383(15)30027-9

[13] Tkatschenko D, Hansen S, Koch J et al (2023) Prevention of surgical site infections in spine surgery: an international survey of clinical practices among expert spine surgeons. Global Spine J 13:2007–2015. 10.1177/21925682211068414 35216540 10.1177/21925682211068414 PMC10556889

[14] Ukay I, Strahm C, Vogt M et al (2023) Antibiotikaprophylaxe / Präemptive Therapie bei offenen Frakturen in der Orthopädie. Swiss Med Forum. 10.4414/smf.2023.09299

[15] Yao R, Tan T, Tee JW, Street J (2018) Prophylaxis of surgical site infection in adult spine surgery: a systematic review. J Clin Neurosci 52:5–25. 10.1016/j.jocn.2018.03.023 29609860 10.1016/j.jocn.2018.03.023

[16] Yusuf E, Steinrücken J, Buchegger T et al (2015) A descriptive study on the surgery and the microbiology of Gustilo type III fractures in an university hospital in Switzerland. Acta Orthop Belg 81:327–332 26280975

[17] EPRD Jahresbericht (2023) EPRD Endoprothesenregister. DE, Deutschland

