# Supplementary material for: Antibiotic prophylaxis in trauma and orthopedic surgery: Current practices across 36 German hospitals and comparison with recently published national guidelines
Source: Unfallchirurgie (Heidelb). 2025 Dec 5;129(2):123–30. doi: 10.1007/s00113-025-01659-7 (PMC12858472; doi:10.1007/s00113-025-01659-7)
Supplement: Supplementary file 1 — The questionnaire used in this study [file 113_2025_1659_MOESM1_ESM.pdf]

Fragebogen „Antibiotikaprophylaxe in Orthopädie und Unfallchirurgie“

1. Welches systemische Antibiotikum wählen Sie in Ihrer Klinik standardmäßig zur Infektionsprophylaxe bei der Osteosynthese geschlossener Frakturen?

2. Wie lange erfolgt die systemische Antibiotikaprophylaxe bei der Osteosynthese geschlossener Frakturen?
   (Bitte Angabe in Stunden oder Einmalgabe)

3. Welches systemische Antibiotikum wählen Sie in Ihrer Klinik standardmäßig zur Infektionsprophylaxe bei offenen Frakturen Gustilo-Anderson Typ I?

4. Wie lange erfolgt die systemische Antibiotikaprophylaxe bei offenen Frakturen Gustilo-Anderson Typ I?
   (Bitte Angabe in Stunden oder Einmalgabe)

5. Welches systemische Antibiotikum wählen Sie in Ihrer Klinik standardmäßig zur Infektionsprophylaxe bei offenen Frakturen Gustilo-Anderson Typ II?

6. Wie lange erfolgt die systemische Antibiotikaprophylaxe bei offenen Frakturen Gustilo-Anderson Typ II?(Bitte Angabe in Stunden oder Einmalgabe)

7. Welches systemische Antibiotikum wählen Sie in Ihrer Klinik standardmäßig zur Infektionsprophylaxe bei offenen Frakturen Gustilo-Anderson Typ III?

8. Wie lange erfolgt die systemische Antibiotikaprophylaxe bei offenen Frakturen Gustilo-Anderson Typ III?
   (Bitte Angabe in Stunden oder Einmalgabe)
9. Welches Antibiotikum wählen Sie in Ihrer Klinik standardmäßig zur Infektionsprophylaxe bei offenen Fingerendgliedfrakturen?

10. Wie lange erfolgt die orale Antibiotikaprophylaxe bei offenen Fingerendgliedfrakturen?
    (Bitte Angabe in Stunden oder Einmalgabe)

11. Welches systemische Antibiotikum wählen Sie in Ihrer Klinik standardmäßig zur Infektionsprophylaxe in der Primärendoprothetik?

12. Wie lange erfolgt die systemische Antibiotikaprophylaxe in der Primärendoprothetik?
    (Bitte Angabe in Stunden oder Einmalgabe)

13. Welches systemische Antibiotikum verwenden Sie bei dorsalen Instrumentierungen an der Wirbelsäule?

14. Wie lange erfolgt die systemische Antibiotikaprophylaxe bei dorsalen Instrumentierungen an der Wirbelsäule?
    (Bitte Angabe in Stunden oder Einmalgabe)

15. Verwenden Sie Vancomycinpulver lokal als Infektionsprophylaxe bei dorsalen Instrumentierungen?
    Ja/Nein
